# Developing an Artificial Intelligence Solution to Autosegment the Edentulous Mandibular Bone for Implant Planning

**DOI:** 10.1055/s-0043-1764425

**Published:** 2023-05-12

**Authors:** Mohammad Adel Moufti, Nuha Trabulsi, Marah Ghousheh, Tala Fattal, Ali Ashira, Sebelan Danishvar

**Affiliations:** 1 Department of Preventive and Restorative Dentistry, University of Sharjah, United Arab Emirates; 2 Brunel University London, United Kingdom

**Keywords:** artificial intelligence, CBCT segmentation, dental implants, implant planning, implantology

## Abstract

**Objective** Dental implants are considered the optimum solution to replace missing teeth and restore the mouth's function and aesthetics. Surgical planning of the implant position is critical to avoid damage to vital anatomical structures; however, the manual measurement of the edentulous (toothless) bone on cone beam computed tomography (CBCT) images is time-consuming and is subject to human error. An automated process has the potential to reduce human errors and save time and costs. This study developed an artificial intelligence (AI) solution to identify and delineate edentulous alveolar bone on CBCT images before implant placement.

**Materials and Methods** After obtaining the ethical approval, CBCT images were extracted from the database of the University Dental Hospital Sharjah based on predefined selection criteria. Manual segmentation of the edentulous span was done by three operators using ITK-SNAP software. A supervised machine learning approach was undertaken to develop a segmentation model on a “U-Net” convolutional neural network (CNN) in the Medical Open Network for Artificial Intelligence (MONAI) framework. Out of the 43 labeled cases, 33 were utilized to train the model, and 10 were used for testing the model's performance.

**Statistical Analysis** The degree of 3D spatial overlap between the segmentation made by human investigators and the model's segmentation was measured by the dice similarity coefficient (DSC).

**Results** The sample consisted mainly of lower molars and premolars. DSC yielded an average value of 0.89 for training and 0.78 for testing. Unilateral edentulous areas, comprising 75% of the sample, resulted in a better DSC (0.91) than bilateral cases (0.73).

**Conclusion** Segmentation of the edentulous spans on CBCT images was successfully conducted by machine learning with good accuracy compared to manual segmentation. Unlike traditional AI object detection models that identify objects present in the image, this model identifies missing objects. Finally, challenges in data collection and labeling are discussed, together with an outlook at the prospective stages of a larger project for a complete AI solution for automated implant planning.

## Introduction

Dental implants are metal screwlike posts inserted in the edentulous (toothless) areas of the jawbone to replace missing teeth. Implants mimic the tooth root and retain the dental prosthesis (tooth crown), offering a fixed alternative to movable dentures that cause inconvenience to wearers. 1 Even for the cases where a fixed bridge can be made on natural teeth, the implants avoid the required invasive preparation of natural teeth and provide a conservative alternative.

For these reasons, implants have become the preferred treatment modality to replace missing teeth. 2 However, before the surgical placement of the implant, it is critical to ascertain that an adequate amount of bone is available to support the implant and to keep a safe distance around the implant to avoid damaging the adjacent vital structures (teeth, nerves, etc.). 1 2 3 A meticulous assessment of the jawbone is usually undertaken on 3D X-rays (cone beam computed tomography [CBCT] images) using a “Digital Implant Planning” software. This manual 3D measurement of the bone volume is technique-sensitive, time-consuming, and subject to human error. 3 4 It depends on the examiner's ability to interpret the images and detect the different anatomical structures, 3 4 which undoubtedly requires the specialist to undergo an intensive and costly training to master the software.

Artificial intelligence (AI) has the potential to replace many tasks presently accomplished by radiologists and surgeons including the detection, characterization, and quantification of anatomical and pathological features. 5 6 7 Thus, AI tools can overcome common problems in health care systems such as work intensity and eye strain during the manual segmentation as well as limited experience of the individual to interpret radiographic images. 2

Recall that AI is a broad term within the discipline of computer science that seeks to replicate human cognitive abilities by creating intelligent entities in the form of software programs. 8 Specifically, “artificial neural networks” are designed to mimic neurons in the human visual system and are arranged in layers akin to networks. They can perform many cognitive tasks such as learning how to recognize objects in images and how to make decisions. 6 AI has found numerous applications in health care, including disease detection, diagnosis, treatment planning, and outcome prediction. 6 Computer vision techniques have assisted in the detection of lung nodules on computed tomography (CT) scans and in diagnosing common lung diseases, segmentation of the pharyngeal airway on CBCT images, and interpretation of breast 3D scans, and have many other applications. 9 10 11 In dentistry, computer vision has been used in recognizing teeth ID on orthopantomogram (OPG), 12 identifying carious lesions on intraoral radiographs, 13 delineation of root canals on CBCT images, and segmentation of inflamed gingiva captured by an intraoral camera. 4

This study aims to develop a computer vision model that can examine CBCT images of the mandibles (lower jaws), identify areas of missing teeth that are candidates to receive dental implants, and delineate the 3D bone volume available for these implants. This process represents the first stage in the digital implant planning undertaken by clinicians that is followed by the selection of the best implant size, direction, and depth that fit the delineated bone segment.

## Materials and Methods

Before commencing the study, an ethical approval was obtained from the University of Sharjah Research Ethics Committee, with the number REC-21-06-08-01, dated July 6, 2021.

### Data Acquisition

A total of 43 CBCT images were extracted from the University Dental Hospital Sharjah using Romexis software. The following inclusion and exclusion criteria were used to select the images and ensure homogeneity in the sample.

- Inclusion criteria:
  - Any case of missing lower teeth, it being single or multiple, anterior or posterior, uni- or bilateral.
  - The edentulous space must be a bounded saddle.
  - The space could have been replaced with a pontic crown as a part of a bridge.
  - Any age or gender.
- Exclusion criteria:
  - Unbounded saddles.
  - Fully edentulous jaws.
  - Edentulous spaces with remaining roots or where the teeth were replaced with implants.

### Data Labeling

**Fig. 1:**
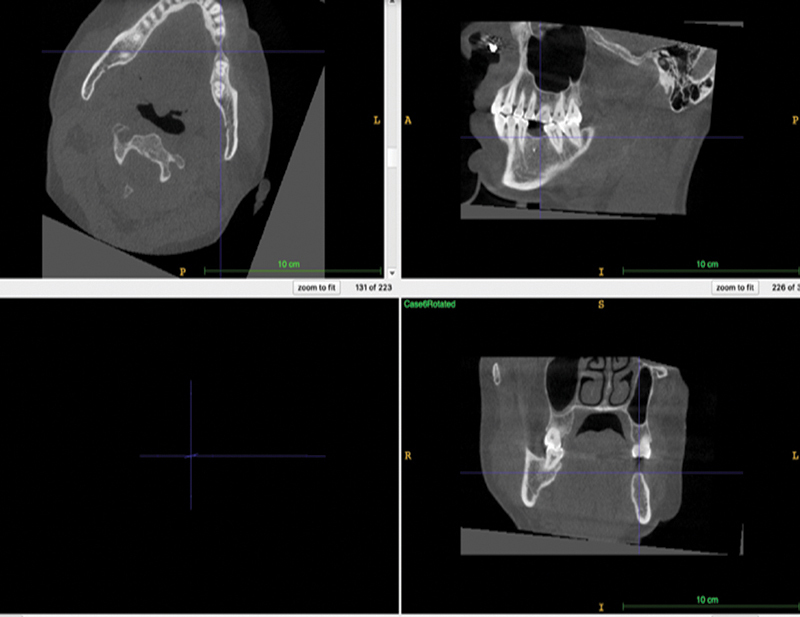
Orientation of the edentulous span in ITK-SNAP software for better visualization of the edentulous area in all three planes.

**Fig. 2:**
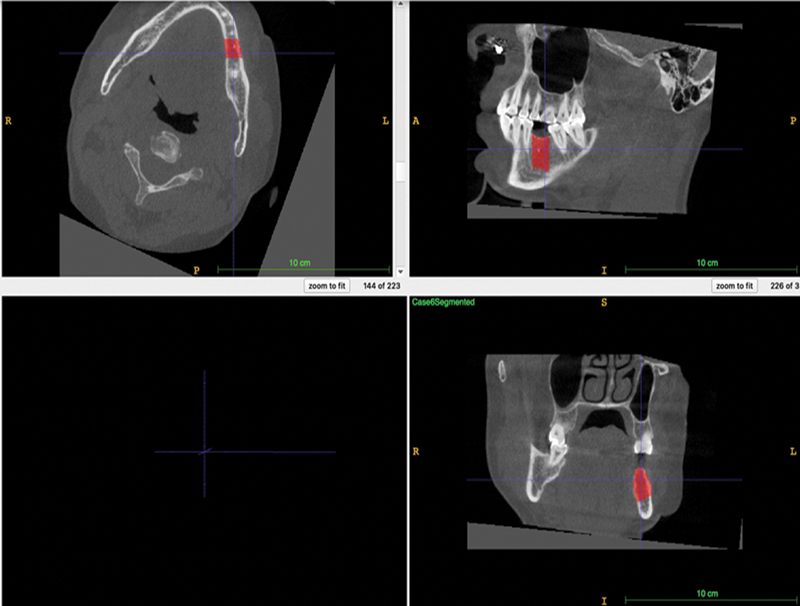
Edentulous span with an overlay mask in *red* (annotation).

The manual segmentation of the edentulous spans was made by two operators using ITK SNAP software version 3.8, which provides semiautomated segmentation. The process comprised two stages:

- *Orientation of the radiograph* : The images were rotated to align the alveolar ridge with the ITK viewer's axis to improve visualization of the ridge ( Fig. 1 ).
- *Segmentation of the edentulous area* : The polygon tool was used to delineate the edentulous span on multiple sections of the coronal view, then the sections were interpolated to cover the entire area and produce a 3D label (mask; Fig. 2 ).

### Quality Assurance

Each of the 43 images was labeled by one of the two student investigators and then verified and approved by the other student and a third investigator (a specialist). All three investigators have undergone intensive training and calibration on the use of the software.

### Model Construction

**Fig. 3:**
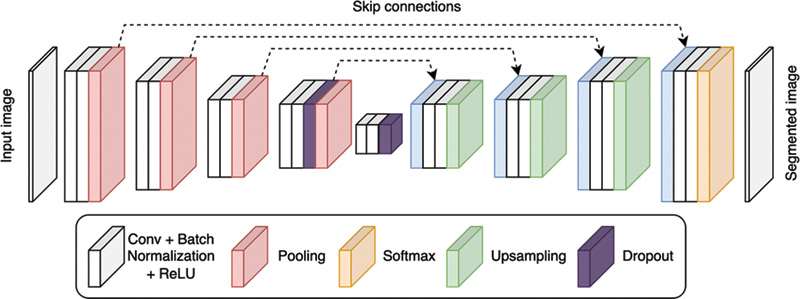
U-net Architecture.

The neural network used was “U-Net,” a type of convolutional neural network (CNN), as shown in Fig. 3 . This network was trained, under supervision, from scratch using our data and the training code was implemented in Python programming language. The enhanced version of U-Net was employed from the MONAI framework, 14 an open-source PyTorch deep learning platform that is freely available and MONAI community supported. The framework allows its developers to build workflows for health imaging training.

Thirty-three images were used for model training and 10 images for model testing. The degree of pixelwise overlap between the manually annotated and model-predicted segmentation was measured using the Dice Coefficient, a score between 0 and 1, with 1 indicating complete overlap.

## Results

As presented in Table 1 , the study sample consisted mainly of lower molars and premolars, with most spans having a single missing tooth. The edentulous areas were unilateral in 75% of the cases, and the male-to-female ratio was 1.68.

**Table 1:** Descriptive analysis of the study sample

Variables			Training ( *n* = 33)		Testing ( *n* = 10)		Total ( *n* = 43)	
Gender	Male	22	5	27	
	Female	11		5		16		
Space	Single	23	3	26	
	Multiple	10		7		17		
Side	Right	10	2	12	
	Left	12		2		14		
	Both	11		6		17		
Number of teeth	Canine	2	0	2	
	Premolar	12		7		19		
	Molar	46		15		61		
Size of edentulous space	1 unit	32	11	43	
	2 units	9		4		13		
	3 units	2		1		3		
	4 units	1		0		1		
Dice similarity coefficient (D.S.C.) average value			0.89		0.78		0.83	

**Fig. 4:**
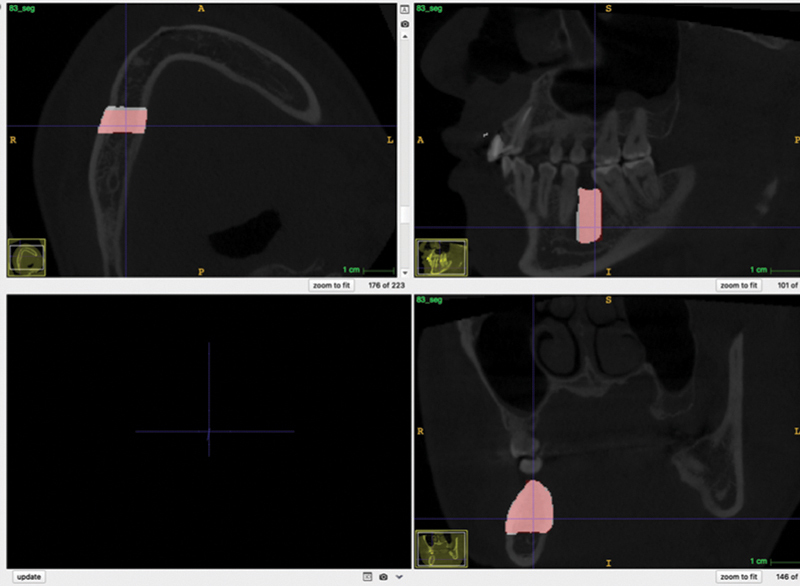
Example of overlap between manual segmentation ( *red* ) and automatic segmentation ( *white* ) on a unilateral case.

**Fig. 5:**
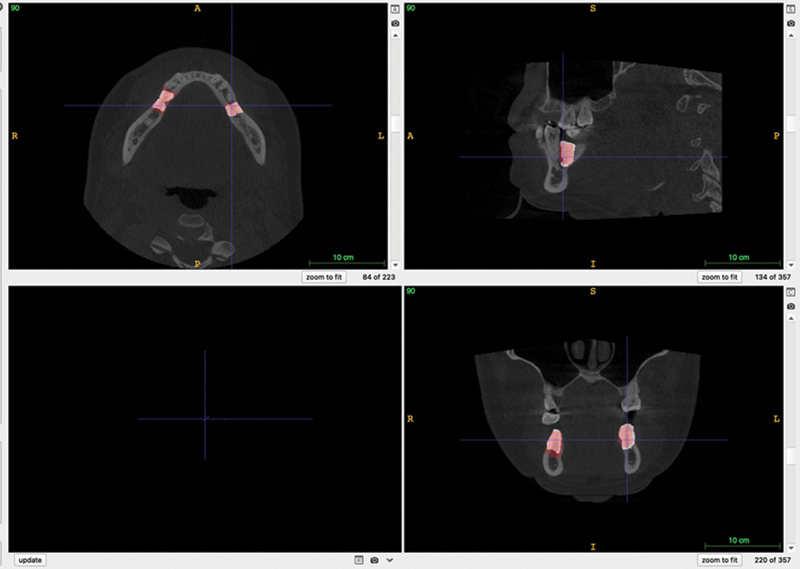
Example of overlap between manual segmentation (red) and automatic segmentation (white) on a bilateral case.

Figs. 4 and 5 show the overlap of manual and automatic segmentation. The dice coefficient ranged between 0.55 and 0.91, with an average value of 0.89 for the training sample and 0.78 for the testing sample. Unilateral cases had a better DSC (0.91) than bilateral cases (073).

## Discussion

In developing the computational model, it was important to select the study sample carefully to achieve generalizability while maintaining reasonable homogeneity in the sample. For this purpose, this phase of the research focused only on the mandibular (lower jaw). Cases with maxillary (upper) missing teeth were excluded since the anatomy and structures surrounding these teeth differ considerably from the mandible. Adding the maxillary sinuses, nasal cavity, and incisive fossa at this stage would have complicated the segmentation process and affected the model's performance. Previous AI studies were unable to detect maxillary anatomical landmarks correctly, thus lowering the success rate to 66.4% when compared to a 97.9% success rate in identifying the mandibular canal. 2 Other studies have reported successful results in identifying the mandibular canal on CBCTs and panoramic radiographs. 3 15 Similarly, in this study, we excluded cases with no anatomical distal boundary (medically known as “distal end extensions” or “unbounded saddles”) as these distal boundaries are usually defined clinically, not radiographically. This was in line with and Taha and Hanbury 12 who reported an inverse correlation between the accuracy of the annotation and the absence of boundaries.

On the other hand, to ascertain generalizability, we selected cases with edentulous areas of various spans, and locations in the mouth (posterior and anterior, unilateral and bilateral). However, the increased variability reduced the model accuracy. Among the above variations, bilaterality had the highest impact on model performance for reasons explained at the end of this section. These observations correspond with a previous report of an inverse correlation between the accuracy of the annotation and the size of the area segmented 12 and number of teeth present. 16 The lower performance might have been in part due to the reduced proportion of bilateral cases in the training sample (33%) compared to the testing sample (60%).

Among the various applications available for image annotations, only a few can view DICOM (Digital Imaging and Communications in Medicine) images. DICOM is the standard for the communication and management of medical imaging information and related data. The ITK-SNAP software has a strong track record in medical research and has been used for the segmentation of numerous structures including thoracic PET tumors, complex-shaped lung tumors, and lateral ventricle. 17 18 19 Other similar software applications include FreeSurfer, Vbm, and Ibaspm. 20 However, ITK-SNAP has the advantage of facilitating a semimanual segmentation, thus making the labeling process faster and easier. When comparing manual delineation to ITK's semiautomatic segmentation, the latter is more accurate, efficient, and reproducible. 18 In dentistry, most contemporary CBCT viewers such as Romexis and Sidexis provide preprogrammed functions for autosegmentation of certain structures. However, these functions are intended for clinical utility such as producing a printable STL model of the jaw. They do not permit exporting the annotated data in a format usable for model training. InvivoDental 6.0 has been used to carry out bone segmentation in both the maxillary and mandibular jaws. 2

The model was developed on a CNN. These deep networks have demonstrated excellent ability for image analysis (computer vision) both theoretically and practically, and they are commonly used to segment medical images. 7 U-Net is an encoder–decoder network architecture, widely used for precise and fast medical image segmentation. 21 Moreover, U-Net has been used with different X-ray modalities such as CBCT and magnetic resonance imaging (MRI). 21 Another network type, probabilistic neural network (PNN), is used for the detection of vertical root fractures in intact and endodontically treated premolar teeth. 22

The accuracy of the segmentation model being developed relates to three qualities, namely, alignment or position of the segmented object, size or volume of the segmented object, and delineation of the boundary, also known as the contour. 12 After training the computational model and fine-tuning its parameters, its image segmentation performance was validated and tested against unseen images. This confirms the applicability and generalizability of the model in day-to-day clinical practice. The segmentation results are compared to the human-segmented images. Different comparison methods are available to assess the model accuracy, including DSC that is particularly useful when evaluating the overlap between two segmented images. 12 23 Other methods of accuracy assessment include the relative volume error (RVE) and the 95% Hausdorff distance. 24 In our study, the overall average DSC value for the testing sample was 0.78, which is slightly lower than that for the training sample (0.83). As noted earlier, this might be due to the higher proportion of bilateral cases in the testing sample (60%) compared to 33% of the training sample.

Our results correlate with another study that reported a slightly higher overlap in alveolar bone segmentation between manual and AI (a DSC average of 85.3%). 4 However, the study mainly focused on segmenting the anterior segment of the jaw, which narrows down the volume that the model needs to process, and overcomes the difficulty of handling curved objects, as discussed below. The study has also used ultrasonography, which limits the generalizability and utility in clinical practice. A recent publication 4 has come to light during the conduction of this study, which had a very similar goal and data type to the present study. The article examined the AI's ability to segment edentulous bone and evaluated the model's accuracy by comparing bone dimensions (height and thickness) to the human measurements. No statistically significant difference was found between the bone height measurements, but no information is provided on the accuracy of bone location, that is, overlap between the human and model's segmentations.

Factors affecting the model accuracy (overlap between the human and model's segmentation) are the following:

- *The challenge of segmentation of distant objects* . ITK provides a semimanual segmentation function by interpolating annotations made on different slices and connecting them to create a 3D mask. In this study, we created the polygons in the coronal view starting from the most distal (posterior) to the most mesial (anterior). To make an accurate polygon delineation of the target area in the slice, the investigator needs to realign the viewer to give an optimum view. However, there is no way to rotate the viewer in ITK. Instead, the image itself has to be rotated to align with the existing axial sections. The rotated image is then exported and reimported for segmentation. This is not only time-consuming but also challenging in bilateral cases where each side of the mandible had a different direction/angulation to the sagittal plane. As such, the image had to be rotated and exported for each side separately, then each export was reimported and segmented alone, and the two images merged computationally. This may explain the reduced accuracy in bilateral cases.
- *The challenge of segmenting unbounded saddles.* As noted earlier, some patients have no teeth at the back of their mouth, which leaves the edentulous space with no posterior boundary. Including these cases in the study sample at this stage of the research would have skewed the results. Further development of the model will address this point since the proportion of this patient cohort is considerable.
- *Accuracy of the boundary delineation.* CBCT images are monochromatic. The different structures are represented with varying shades of gray, which reduces the accuracy of autosegmentation of the software (ITK). A meticulous review and manual correction of the erroneous areas is mandatory, but it is time-consuming.
- *Time and manpower.* In addition to the above-noted challenges that require considerable time to solve, it is important to keep in mind that segmenting medical images requires field specialists in the first place. Nonspecialist image annotation professionals who are dedicated to these tasks can only provide segmentation for general purposes (e.g., cars and everyday images). The availability of these specialists is a considerable limiting factor for the sample size discussed next.
- *The study sample size.* Having a sufficient sample size is required for good model training. A large dataset is a prerequisite to start with. However, even when this is available, the stringent case selection criteria, which aim to improve the model's accuracy, reduce the number of available cases significantly. This in turn affects the model's accuracy!
- *Accuracy of the concept of ground truth.* The model training is based on the concept that human annotation is the *truth* , and aims to achieve an identical segmentation to the human, at its best. However, when both segmentations do not fully overlap, one must consider if the human annotation was inaccurate in the first place. And here we do not refer to erroneous inaccuracy, but an intentional one. In dentistry, it is often observed that the tooth posterior to the edentulous space tilts toward the space, making the space narrower at the superficial (top part, occlusal part) part of the bone than its deep part (root side). The result is a trapezoid shape of the edentulous bone, with a wide base. Implants, on the other hand, are mostly cylindrical, and their size selection is determined by the bone available in the narrowest space, that is, the top. For this reason, we manually segmented the bone as a cuboid shape with parallel anterior and posterior boundary slices. We noticed that the model segmented all bone available between the teeth, resulting in a trapezoid area slightly larger than the cuboid. The model correctly identified the available volume of bone between the adjacent teeth but did not accurately match the human segmentation.
- *Length of the saddle.* The highest and lowest DSC values in the testing cases were 0.91 and 0.55, respectively. When comparing both cases, it was noted that the latter presented with the longest edentulous bilateral span in the sample. Kanuri et al 16 and Taha et al 12 supported this finding in their study when they stated that the value decreases with a smaller number of present teeth and larger areas to be segmented,.
- *Using one CBCT device.* To generalize our results and use the developed program/code in different settings, the model training should be performed on images from different imaging machines and using diverse data from other hospitals.
- The model itself was developed from scratch and did not depend on learning transfer.

### Strengths and Limitations

Relative to the small sample size, the model has achieved a good accuracy (>90%) in segmenting unliteral cases, which represent the majority of patients with missing teeth. This might be attributed to the highly accurate segmentation of the images by the investigators, which was the result of the calibration and quality assurance processes. The model itself was purpose-built and did not utilize learning transfer. Enlarging the study sample size, widening the clinical variations, and improving the balance between unilateral and bilateral cases in the training section can significantly improve the results. The current model is limited to the mandible and to the bounded saddles, which limits its generalizability at this stage.

## Conclusion

Within the limitation of this study, the model has achieved a good level of accuracy in segmenting edentulous bone areas compared to the human investigators. This automation of bone assessment on CBCT images has the potential to significantly reduce the time and associated cost of implant treatment. Future development of the model is required to improve generalizability and accuracy in challenging conditions.
